# Redox Potential of Antioxidants in Cancer Progression and Prevention

**DOI:** 10.3390/antiox9111156

**Published:** 2020-11-20

**Authors:** Sajan George, Heidi Abrahamse

**Affiliations:** 1 School of Biosciences and Technology, Vellore Institute of Technology, Vellore, Tamil Nadu 632014, India; sajan.george@vit.ac.in; 2 Laser Research Centre, University of Johannesburg, Doornfontein, Johannesburg 2028, South Africa

**Keywords:** redox potential, antioxidants, cancer

## Abstract

The benevolent and detrimental effects of antioxidants are much debated in clinical trials and cancer research. Several antioxidant enzymes and molecules are overexpressed in oxidative stress conditions that can damage cellular proteins, lipids, and DNA. Natural antioxidants remove excess free radical intermediates by reducing hydrogen donors or quenching singlet oxygen and delaying oxidative reactions in actively growing cancer cells. These reducing agents have the potential to hinder cancer progression only when administered at the right proportions along with chemo-/radiotherapies. Antioxidants and enzymes affect signal transduction and energy metabolism pathways for the maintenance of cellular redox status. A decline in antioxidant capacity arising from genetic mutations may increase the mitochondrial flux of free radicals resulting in misfiring of cellular signalling pathways. Often, a metabolic reprogramming arising from these mutations in metabolic enzymes leads to the overproduction of so called ’oncometabolites’ in a state of ‘pseudohypoxia’. This can inactivate several of the intracellular molecules involved in epigenetic and redox regulations, thereby increasing oxidative stress giving rise to growth advantages for cancerous cells. Undeniably, these are cell-type and Reactive Oxygen Species (ROS) specific, which is manifested as changes in the enzyme activation, differences in gene expression, cellular functions as well as cell death mechanisms. Photodynamic therapy (PDT) using light-activated photosensitizing molecules that can regulate cellular redox balance in accordance with the changes in endogenous ROS production is a solution for many of these challenges in cancer therapy.

## 1. Introduction

Reactive oxygen species (ROS) are the intermediates of the metabolic processes in organelles such as endoplasmic reticulum, mitochondrial respiratory complex, peroxisomes, and processes such as detoxification of xenobiotics, oxidation of fatty acids etc. [1]. Under normal conditions, ROS are produced in picomolar concentrations and any amount of excessive ROS gets neutralized by the antioxidant system of the body. However, sometimes, there will be an impairment or imbalances in this process leading to the accumulation of free radicals, complicating diseases such as cancer, cardiovascular diseases, diabetes, etc. Metabolically active cells develop higher amounts of free radicals, which can alter cellular homeostasis leading to the development of malignancy and metastasis. The association between cardiovascular diseases, myocardial oxidative stress, and the beneficial role of antioxidants in preventing atherosclerosis is quite popular in clinical medicine. Presence of oxide radicals predisposes the oxidation of low-density lipoproteins for the formation of atherosclerosis resulting in blockage of arteries [2]. A higher level of ROS is also responsible for various pathologies viz. aging, endothelial dysfunction, insulin resistance, diabetic nephropathy, etc. Many of these harmful effects induced by oxide radicals on the cardiovascular diseases are alleviated by the supplementation of antioxidants [3]. The intake of antioxidants may help the diabetic patients to reduce the free radical overload in the system and overcome the hyperglycaemic state [4].

Growing cells adjust endogenous ROS for outcomes such as cellular senescence, apoptosis, and necrosis. Approximately 2% of the oxygen entering in the body by respiration is converted into free radicals by cellular and mitochondrial processes under normal physiological conditions [5]. Living cells synthesize nitric oxide radicals (NO^•^) that function as an endothelial relaxing factor and neurotransmitter produced by the nitric oxide synthase (NOS). Nitric oxide (NO^•^) and superoxide (^•^O_2_) radicals are converted into alkoxyl (^•^RO) radical, peroxyl (^•^ROO) radical, hydroxyl (^•^OH) radical, and singlet oxygen (^1^O^−^_2_) anions by a series of biochemical reactions. Further, these powerful oxidizing radicals are converted into molecular oxidants like hydrogen peroxide (H_2_O_2_) and peroxynitrate (ONOO^−^) anions, as well as hypochlorous acid (HOCl), which may act as a source of ROS [6]. ROS can induce oxidation of cell membranes due to the presence of high concentration of fatty acids in the lipid bilayer. In fact, ROS can cause the lipid peroxidation of lipid membranes, forming lipid peroxyl (^•^LOO) radicals, resulting in the cross linking of membrane proteins and alterations in membrane fluidity. It can also result in the formation of lipid-DNA adduct and lipid-proteins that can harm the physiological functioning of the cell [7]. ROS interacts with proteins resulting in changes to the tertiary structures, proteolytic degradation, fragmentation, peroxidation, protein-protein cross linkages as well as the formation of carbonyls viz. aldehydes and ketones. It may attack the sugar moieties in the DNA strand producing sugar peroxyl (^•^ROO) radicals causing breakage of strands and interact with DNA causing modification of DNA bases. The net result of these oxidative damages on the genetic material is ageing, mutagenesis and death of the cells [8].

Mitochondrial energy production is achieved as oxygen is reduced along the electron transport chain (ETC) located on the inner membrane leading to the production of oxygen free radicals. Several species of oxide free radicals are generated from singlet oxygen (^1^O^−^_2_) anion, which is the most reactive ROS by a single step electron transfer process. One electron reduction of the singlet oxygen (^1^O^−^_2_) anion generates superoxide (^•^O_2_) radicals and a further reduction of one electron from these superoxide (^•^O_2_) radicals produces hydrogen peroxide (H_2_O_2_) [9]. The primary role of the oxide free radicals is the destruction of microorganisms as performed by the white blood cells, especially neutrophils through the nicotinamide adenine dinucleotide phosphate (NADPH)-oxidase activity [10]. They are also responsible for the intercellular and intracellular signalling necessary for various physiological activities leading to the proliferation and differentiation of cells [11]. Often, low levels of ROS activate cellular glutathione by triggering the antioxidant response element (ARE) producing proliferative effects on a variety of cell types, whereas concentrations above 100 µM result in the activation of cell death pathways (referred as biphasic response) [12]. During hyperoxia or hypoxia and treatment using ionizing radiations, a large quantity of oxide free radicals are gemerated that can harm the cells. Together, superoxide (^•^O_2_) radicals and hydrogen peroxide (H_2_O_2_) increase DNA replication and the cell cycle to act as mitogen predisposing cells to develop cancer [13,14].

## 2. Endogenous Antioxidants

Antioxidants originate from either endogenous or exogenous sources. The endogenous antioxidants consist of enzymatic antioxidants viz. catalase, glutathione (GSH) dependent enzymes viz. glutathione peroxidases (GPx), glutathione reductases, glutathione S-transferases, superoxide dismutase (SOD), thioredoxin etc. and non-enzymatic antioxidants, which are sub-divided as metabolic and nutrient antioxidants. Metabolic antioxidants consist of bilirubin, coenzyme Q10, GSH, L-arginine, melatonin, uric acid, etc. Nutrient antioxidants consist of α-lipoic acid, β-carotenoids, flavonoids, polyphenols, and vitamins C and E, etc. All these antioxidants have the unique capability of scavenging and mopping up of free radicals by donating their own electrons and neutralizing the electrical charge [15]. Ideally, antioxidants should readily be synthesized, chelate redox metals, and eliminate free radicals helping the metabolically active cells to achieve homeostasis.

Depending to their action on cellular free radicals, endogenous antioxidants may be preventive or scavenging and often performing radical-induced damage repair. Accordingly, they are sub-divided into the following categories. First line of defense: They include catalase, GPx and SOD, which act on hydrogen peroxides (H_2_O_2_), alkyl hydroperoxidases (ROOH) and superoxide (^•^O_2_) radicals respectively to prevent the formation of free-radicals and ROS. Second line of defense: They are antioxidants that scavenge active free radicals by donating electrons to inhibit chain initiation and propagation. Later on, these scavenging antioxidants become free radicals of less potential, which are easily neutralized by the first-line antioxidants viz. ascorbic acid, uric acid, glutathione, ubiquinol, vitamin E, etc. Third line defense: These are de novo enzymes that repair the damages caused by free radicals on cellular biomolecules such as DNA, protein, and lipid to restore cellular functions viz. nucleases, polymerases, peptidases, lipases. Fourth line defense: These antioxidants are produced as a part of cellular adaptation and utilize signals required for free radical production [16].

Catalase: Hydrogen peroxide (H_2_O_2_) is harmful to cells and it is converted to extremely reactive hydroxyl radical (^•^OH) in the presence of cuprous and ferrous ions by a Fenton reaction. Catalase, a first line antioxidant enzyme located in the peroxisomes (catalase is absent in mitochondria of mammalian cells), utilizes either iron or manganese as cofactor for converting hydrogen peroxide (H_2_O_2_) into water and molecular oxygen (O_2_) [17] (Equation (1)). The enzyme functions in two steps (i) oxidation of heme to an oxyferryl species by hydrogen peroxide (H_2_O_2_) and (ii) generation of a porphyrin cation radical following oxidation of iron and the porphyrin ring. Catalase consists of four subunits molecular weight 240 Kilodalton encoded by the gene *ctt1* located in chromosome 11. Thus, it exists as a tetrameric protein with each subunit containing a heme group and a molecule of NADPH. Genetic polymophisms as well as mutations of *ctt1* is responsible for the onset of various diseases related to the alterations in the cellular oxidative status including cancer [17,18]. However, there is contradictory evidence concerning the role of catalase enzyme on neoplasms of various types of tissues [19].
(1)
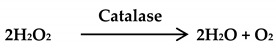



Glutathione (GSH): A major antioxidant defence molecule existing in all cellular compartments, which is composed of amino acids cysteine, glutamine, and glycine. It is one of the key antioxidants which play a greater role in the synthesis of nucleic acids, proteins as well as in the detoxification of xenobiotics [20]. GSH is synthesized mainly by the liver and converted as oxidized glutathione by the enzymes glutathione peroxidase (GPx) as well as glutathione reductase to neutralize the free radicals and resides in the cytoplasm of all metabolically active cells. GSH performs amino acid transport across plasma membranes, functions as a co-factor for several enzymes, regenerate vitamin C and E after utilization as antioxidants and scavange various oxide radicals in the cell [21]. In fact, the sulfhydryl group present in the glutathione allows it to function as a cellular antioxidant. A decline in the glutathione/glutathione disulphide (GSH/GSSG) ratio is responsible for the increase in oxidative stress leading to carcinogenesis (Equation (2)). Thus, an increased GSH/GSSG ratio due to the elevated GSH levels is desirable for reducing oxidative stress and preventing cancer progression, facilitating cells to achieve a state of homeostasis [22]. Further, decreasing the levels of extracellular cysteine (two linked cysteine molecules) uptake or the use of glutaminase inhibitors is a therapeutic strategy in cancer cells to induce excessive ROS and cell death [23].
(2)
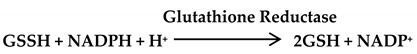



Glutathione Peroxidase (GPx): In cellular mitochondria, the first line antioxidant, GPx, converts hydrogen peroxide (H_2_O_2_) into water and fatty acid peroxides into alcohols through H_2_O_2_-mediated oxidation of reduced glutathione (GSH) [24]. It uses glutathione reductase and NADPH to reduce oxidized glutathione (GSSG) back to GSH. GPx exist as many as eight different GPx enzymes encoded by different chromosomes, which may or may not be associated with the cofactor selenium. Thus, GPx are classified as (i) Selenium-dependent (also known as selenocysteine peroxidase) and (ii) Selenium-independent based on the catalytic mechanism and the number of subunits. It is the selenium-dependent GPx that is capable of oxidizing GSH, while catalyzing the conversion of hydrogen peroxide (H_2_O_2_)or organic peroxide to alcohol or water [25] Equation (3). Deficiency of GPx will lead to excessive oxidative stress causing damages to cellular proteins and membrane lipids. Particularly, GPx1 is attributed with the prevention and progression of cancer as well as cardiovascular diseases [26].
(3)
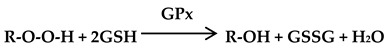



Heme oxygenase: Heme oxygenase-1 is the enzyme for the degradation of heme into biliverdin, bilirubin, carbon monoxide (CO) and free iron (Equation (4)). It is responsible for the adaptive responses of the physiologically active cells during fluctuations in the cellular oxidative stress [27]. The function and expression of heme oxygenase is influenced by various signaling pathways, transcription factors, and the metabolic status of the normal as well as diseased cells [28].
(4)
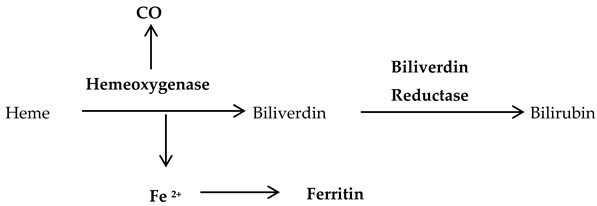



Peroxiredoxins: These are a family of six isoenzymes with antioxidant action on alkyl hydroperoxides (ROOH), hydrogen peroxide (H_2_O_2_) and the peroxynitrite (ONOO^−^) anion. Mitochondrial and cytosolic peroxiredoxins can detoxify hydrogen peroxide (H_2_O_2_) to water while undergoing oxidation of their active-site cysteines (Equation (5)). This oxidized peroxiredoxins is reduced again by NADPH, which is synthesized from thioredoxin by the action of thioredoxin reductases [29]. Peroxiredoxins is upregulated by Nuclear factor erythroid 2-related factor 2 (Nrf2), a transcription factor, which controls the regulation of several antioxidant genes. It also interacts with several other transcriptional regulators, including *c-Myc* oncogene, to suppress breast cancer development [30].
(5)
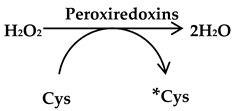



Superoxide Dismutase (SOD): Located in the cytosol and mitochondria, SOD catalyses the dismutation of two molecules of superoxide (^•^O_2_) radicals to hydrogen peroxide (H_2_O_2_) and oxygen molecule (O_2_) (Equation (6)). It is a metalloenzyme requiring a metal cofactor for catalytically deactivating and converting the powerful superoxide (^•^O_2_) radicals or singlet oxygen (^1^O^−^_2_) anion to less hazardous molecules [31]. On the basis of the type of metal ion as co-factor three forms of SOD are present in humans: cytosolic Cu, Zn-SOD, mitochondrial Mn-SOD, and extracellular-SOD [32]. Indeed, the function and location of SOD are modified based on these cofactors, which include (i) Cu/Zn-SOD found in the cytosol as well as peroxisomes of the eukaryotic cells, (ii) Mn-SOD found in the mitochondria of eukaryotes and prokaryotes, and (iii) Fe-SOD found in the prokaryotes as well as the chloroplasts of plants [16]. The deficiency of SOD occurs with age and will lead to an increase in the free radicals causing diseases viz. amyotrophic lateral sclerosis (ALS), cerebral vascular hypertrophy, vascular dysfunction associated with hyperhomocysteinemia, as well as conditions like myocardial injury and perinatal death in mice models [33,34,35].
(6)
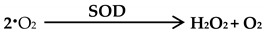



Thioredoxins: Thioredoxin-1 is located in the cytoplasm as well as nucleus, while, Thioredoxin-2 is located in the mitochondria. These antioxidant molecules possess two cysteine active sites to serve as hydrogen donors to the thioredoxin-dependent peroxide reductases towards reducing oxidized proteins and protecting cells (Equation (7)). Also, they possess a disulphide bond and have two redox-active cysteins within a conserved active site (Cys-Gly-Pro-Cys) [36]. Under homeostasis, the reduced form of thioredoxin-1 binds to the Apoptosis signal-regulating kinase 1 (Ask1), predisposing the latter to the ubiquitination and degradation [37]. Interestingly, thioredoxin along with GSH antioxidant pathways synergize for the total inhibition of oxidative stress, which may turn out to be beneficial for cancer growth and expansion [38].
(7)
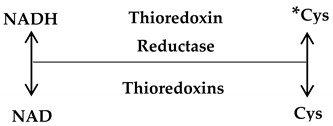



## 3. Exogenous Antioxidants

Several of the exogenous antioxidants are natural compounds, which are hydrogen donors or quenchers of singlet oxygen (^1^O^−^_2_) anion. They are capable of removing free radical intermediates or delaying oxidative reactions. Natural antioxidants from diets include vitamin A, C, E, and various phytoingredients that function differently to promote cell cycle arrest and death of cancer cells. Antioxidants from plant sources, especially carotenoids, flavonoids, phenols, and vitamins have been shown to suppress early and late stages of carcinogenesis [9]. These reducing agents exert anticancer effects via several modalities, including alterations in cell signalling, changes in the cell cycle progression and, modulation of enzymatic activities [15]. Understanding these mechanisms of action of antioxidants is critical in developing and using novel antioxidants in cancer therapy. 

Vitamin A is generated from β-carotene and a vital antioxidant against systemic diseases, especially coronary heart diseases due to its ability to reduce the circulating low-density lipoproteins [39]. Carotenoids are lipid soluble antioxidants existing as α or β isoforms on membranes as lipoproteins and scavenge singlet oxygen (^1^O^−^_2_) as well as peroxyl (^•^ROO) radicals [40]. Vitamin C (ascorbic acid) is a well-known water-soluble free radical scavenger capable of regenerating vitamin E (α-tocopherol) in lipid membranes using reducing equivalents or in combination with GSH (Equation (8)). It is important to note that ascorbic acid is converted to ascorbate radicals during lipid peroxidation, and then, two molecules of ascorbate radicals combine to produce ascorbate and dehydroascorbate. Ascorbic acid is again regenerated from dehydroascorbate by the addition of two electrons in the presence of oxidoreductase enzyme [15]. The lipid soluble vitamin E exerts its antioxidant activity by terminating lipid peroxidation of cell membranes as well as low-density lipoproteins. Thus, vitamin E protects cells from lipid peroxidation with a structural role in stabilizing membranes. The regeneration of vitamin E occurs at the aqueous interface with ascorbates, glutathiones, or urates. Alternatively, it may be oxidized to form tocopherol quinone or dimerize to remain stable in the cell [41].
(8)
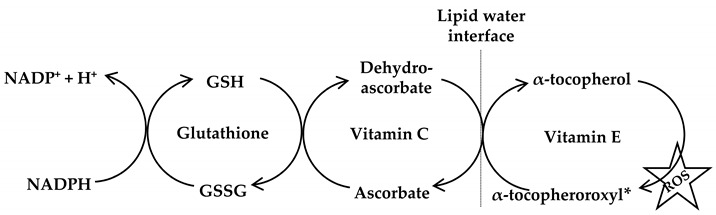



The natural antioxidant, α-lipoic acid, is a thiol antioxidant with metal-chelating (helps to remove toxins) and antiglycation properties. The active form of α-lipoic acid is dihydroxy lipoic acid capable of increasing glutathione and vitamin C or E levels in the cytosol. Flavonoids are benzo-γ-pyrone derivatives consisting of phenolic and pyrane rings, capable of chelating redox-active metals viz. copper and iron and inhibiting lipid peroxidation by scavenging peroxyl (^•^ROO) radicals. In rare situations, flavonoids can influence cell signalling and exhibit pro-oxidant activity when the concentration of hydroxyl groups increases [42]. Conversely, melatonin (N-acetyl-5-methoxytryptamine) is secreted from the pineal gland in the brain to function as a hormone capable of stimulating several other antioxidant and prooxidant enzymes [43]. 

Synthetic antioxidants are substances that function by inhibiting the production of ROS enzymes, sequestration of metal ions and scavenging of free radicals in the biological systems. For example, allopurinol and pyrazolopyrimidines are inhibitors of xanthine oxidase enzyme, desferrioxamine is an iron chelator that can prevent the production of oxide radicals, dimethyl thiourea and butylphenylnitrone are scavengers of hydroxyl (^•^OH) radical. N-acetyl cysteine is a potent antioxidant with the capacity to deacteylate and generates cysteine, which is required for the synthesis of GSH [44]. The most commonly used synthetic antioxidants in the food industry are butylated 4-hydroxytoluene and butylated 4-hydroxyanisole. New synthetic antioxidants are tested and used in cancer therapy despite the concerns regarding their efficacy and safety [5].

## 4. Redox Potential of Antioxidants

Living cells experience ‘oxidative stress’ when the number of free radicals exceeds the capacity of antioxidants to balance and neutralize the excessive levels of free radicals. The presence of two unpaired electrons in separate electronic orbitals renders oxygen more susceptible for free radical formation by stepwise addition of electrons. These oxygen radicals play a fundamental role in oxidative stress and damage in living cells [16]. Thus, the free radical induced cellular toxicity is a consequence of the generation of oxygen radicals that give rise to ROS. There are several important sources of free radical formation viz. mitochondrial ETC, oxidation of endoplasmic reticulum, chemotherapy, radiations, and a wide range of enzymatic activities in the cell. A rise in the free radicals can lead to pathological conditions, when it oxidizes and damages the cellular components [14,45]. Consequently, there will be an increase in the level of oxidation of several biomolecules and cellular structures leading to disease conditions.

A major consequence of the excess oxide radicals is the damages imparted to various amino acids of the cell, especially in the presence of the trace metals ions, Cu^2+^ and Fe^2+^. Moreover, these free radicals are capable of lipid peroxidation and oxidizing free fatty acids leading to physiological and metabolic changes to cells [19]. Antioxidants might be useful in controlling and even reducing this ‘oxidative stress’ that might result in an increased efficacy of anti-cancer drugs. Redox status is defined as the reduction potential of all antioxidant molecules in the cellular constituents or biological fluids. In fact, redox balance is maintained towards a state of negative redox potential values due to the activity of cellular homeostasis mechanisms. This cellular redox balance may be disturbed due to changes in physiological conditions and when there is reduction in the levels of cellular antioxidants [14]. The net result can be manifested as functional damages to health or various disease conditions, including cancer.

Antioxidant molecules exists as either large (Catalase, GPx, SOD) or small (carotinoids, GSH, vitamins C and E) capable of controlling the redox balance in malignant cells [15]. So, one of the strategies for inducing tumor reduction is targeting the redox metabolism by increasing the antioxidant capacity of the cancer cell. For instance, the oxidized form of vitamin C (dehydroascorbic acid) enters into metabolically active cells through glucose transporters and gets reduced into ascorbic acid by GSH (the oxidized GSSG is reduced to GSH by NADPH-dependent glutathione reductase). Water soluble antioxidants, especially vitamin C rely on the reducing equivalence of NADPH. Therefore, administration of high doses of vitamin C may deplete cellular NADPH pool causing an increase in the cellular oxidative stress [46]. In fact, the entry of the small molecule antioxidant, dehydroascorbic acid, which is the oxidized form of vitamin C, is higher into cancer cells as the uptake of glucose is much higher. Alongside, cancer cells have also developed a higher protective effect to these antioxidants as their survival mechanism [47,48].

**Figure 1:**
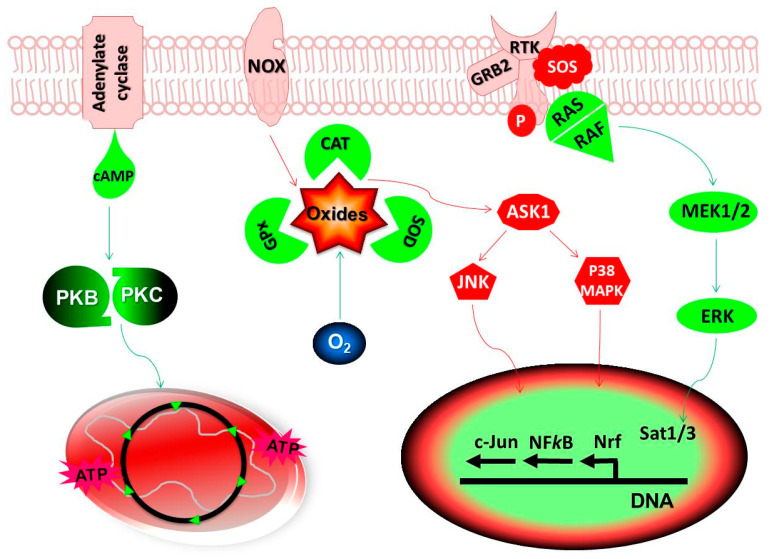
Redox potential of antioxidants: In healthy cells, oxide free radicals generated from mitochondria and cell membrane NADPH Oxidases (NOX) are detoxified by the endogenous antioxidants, catalases (CAT), Glutathione Peroxidases (GPx) and Superoxide Dismutases (SOD). A constitutively low level of Reactive Oxygen Species (ROS) stimulate Protein Kinases (PK) B/C for maintaining cellular homeostasis while, higher levels can result in the change in mitochondrial membrane potential leading to apoptosis or cell death. Cancer cells have higher levels of oxidative stress as compared to the normal cells allowing them to activate several signaling pathways and transcription factors. Any imbalances in the redox-sensitive signaling pathways, Mitogen Activated Protein Kinases (MAPK) and PK B/C may result in the tumor growth. The Ras-Raf-MEK-ERK pathway (also known as the MAPK/ERK pathway) is the signaling cascade from cell membrane receptor complex to the nucleus. During excessive oxidative stress, Apoptosis Signal-regulating Kinase 1 (Ask1) induces apoptosis by activating c-Jun N-terminal Kinases (JNK) and p38 MAPK pathways. It may also lead to the inactivation and proteolytic degradation of PK molecules leading to the initiation of apoptosis or cell death signaling. The redox regulation of cellular oxidative stress is achieved either by activation or repression of the antioxidant genes through several transcription factors, acting individually or in combination. Objects in RED show the pathways activated directly by oxides radicals and the objects in GREEN indicate pathways regulating the cellular antioxidant mechanisms. Abbreviations: cAMP, cyclic Adenosine Monophosphate; ATP, Adenosine Triphosphate; ERK, Extracellular Signal-regulated Kinases; O_2_, oxygen.

In healthy cells, Ask1 induces apoptosis during excessive oxidative stress by mediating the activation of both c-Jun N-terminal Kinase (JNK) and Mitogen Activated Protein Kinases (MAPK) pathways (Figure 1). The primary role of p38 MAPK is the inhibition of tumor growth, although its prooncogenic role is also identified in different cancer cell lines [49]. Usually, healthy cells receive stimulation by cell surface receptor binding of various growth factors that signals through phosphatidylinositol 3-kinase (PI3K)/Akt/mammalian target of rapamycin (mTOR) pathways. This results in the normal levels of glycolytic flux and fatty acid synthesis, leading to the onset of anabolic pathways and production of bioenergy for cellular activities [50]. However, tumor cells hold mutations in the PI3K/AKT/mTOR signaling network causing aberrant activation of anaplerotic pathways leading to the excessive production of Kreb’s cycle intermediates, which make entry from various sites in the Kreb’s cycle for massive energy output [51]. During hypoxia, the PI3K/Akt signaling activates NADPH-oxidase increasing oxidative stress leading to the increase in genomic instability and onset of tumor growth.

An imbalance in the cellular homeostasis activates several genes and transcription factors leading to the increase in the production of ROS viz. cycloxygenase-2, NOS, nitric oxide-2, xanthine oxidoreductases etc. Activation of nuclear factor-κB (NF-κB) may lead to the upregulation of several genes responsible for the angiogenesis, proliferation and transformation of cells leading to the onset of malignancy and metastasis as observed in the colorectal cancer and in hepatocellular carcinomas [52,53]. Altogether, the activity of NFκB is dependent on the level of oxidative stress as well as tumor and organ type. On the contrary, activation of NFκB also lead to expression of antioxidant genes encoding catalase, glutathione S-transferases, GPx, heme oxygenase and various types of SOD [54,55]. Excessive ROS in tumor cells is also responsible for the activation of antioxidant genes and other transcription factors viz. Activator protein 1 (AP-1), Nrf2, and p53. In fact, tumor cells possess higher level of antioxidant enzymes than the normal cells for scavenging excessive free radicals produced during rapid proliferation. These enzymes prevent free radical induced activation of cell death pathways viz. JNK and p38 MAPK [19]. Perhaps, this makes the cancer therapy using additional antioxidants questionable.

The tumor suppressor p53 gene can activate either antioxidant or prooxidant responses. It is capable of inducing expression of antioxidants, Mn-SOD, and heme oxygenase-1 by directly binding to their gene promotors leading to cell survival mechanisms [56,57]. In fact, NAC, which increases the GSH, is found to enhance tumor growth when administered with vitamin E by reducing the expression levels of p53 and depleting ROS [58]. The p53 is also capable of altering the expression of p53-Inducible Genes 1–13 (PIG1–13) that encodes redox-active proteins and TP53-induced Glycolysis and Apoptosis Regulator (TIGAR) to block glycolysis and shuttling of metabolites to Pentose Phosphate Pathway (PPP) [59,60]. Thus, p53 increases PPP-mediated NADPH production for the reduction of GSSG to GSH in the presence of the enzyme glutathione reductases. Moreover, p53 acts on the glutaminase-2 to convert glutamine to glutamate, which is used for generating GSH by modification of cellular metabolism [61]. On the contrary, ROS-generating enzymes viz. quinone oxidoreductases, proline oxidase, and redox active proteins, are upregulated by the stress-induced p53 activation [19]. The p53 induced overproduction of ROS may also occur by the redox-active proteins viz. Bax and Puma, resulting in changes to the mitochondrial membrane permeability [62]. The loss of p53 along with the activation of oncogenes, *c-Myc* and *K-ras* stimulates transcriptional regulation of metabolic genes, which increases glycolytic flux to promote anabolism and expansion of the tumor mass [63].

Tumor cells often increase their antioxidant potentials by activating the transcription factor Nrf2 following disruption with its binding partner Kelch-like ECH-associated protein 1 (Keap1) [64]. In fact, the harmful effects of oxide radicals are neutralized by Keap1-Nrf2 signaling cascade, where Keap1 acts as a sensor and Nrf2 act as a transcription factor for the key antioxidant enzymes. Nrf2 remains bound to Keap1 and sequestrated in the cytoplasm for the regulation of antioxidants, multi-drug resistance associated protein transporters as well as drug metabolizing enzymes. During oxidative stress conditions, Nrf2 is detached from Keap1 and translocated into the nucleus, where it binds to ARE located in the regulatory elements of target genes after dimerization with small Maf (musculoaponeurotic fibrosarcoma) protein. Following activation, Nrf2 induces transcription of enzymes for GSH synthesis as well as antioxidant proteins. Further, Nrf2 activates enzymes for increasing the cytosolic NADPH levels by regulating the serine biosynthesis pathway for maintaining the redox potential during hypoxia. Further, thioredoxin reductase 1 also regulates the activity of Nrf2 for enhancing the redox potentials of cells [65,66]. Overall, Nrf2 activation results in the expression of cytoprotective or stress-responsive enzymes viz. catalase, glutathione S-transferases, glutathione reductases, GPx, heme oxygenase-1, SOD, thioredoxin, and quinone oxidoreductases [67,68]. Not surprisingly, mutations in the *Nrf2* and *Keap1* are responsible for the alterations in the expression of wide range of antioxidant/oxidant genes that favor either cancer progression or prevention (Table 1).

Compelling evidences suggest that protein Kinase C isoenzymes may promote either tumor progression or prevention. Excessive ROS can result in the inactivation by proteolytic degradation and the modification of the enzymatic structure of protein kinase C while, low levels of its stimulation is required for cellular homeostasis [82]. Excessive ROS will also result in the inactivation of tumor suppressor gene PTEN (phosphatase and tensin homolog deleted on chromosome 10), which has a role in reducing the oxidative stress in malignant cells. This will increase the prooncogenic tyrosine kinase receptor activity through PI3K/Akt pathway resulting in the progression of tumor growth by inhibition of apoptosis [83]. The inactivation of PTEN may also increase the secretion of matrix metalloproteinases as well as telomerase enzyme activity for EMT, promoting the growth and metastasis of cancer [84].

An elevated level of oxide radical is the hallmark of cancer, although, an excessive production of oxidative stress lead to cell death. Low to medium levels of free radicals can reversibly oxidize the cysteine residues of cellular proteins by adaption to metabolic stress. However, when the oxidative stress increases, excess hydrogen peroxide (H_2_O_2_) is converted to hydroxyl radical (^•^OH) that can damage various cellular components [19]. The capacity of invasiveness and the ability to metastasize is also dependent up on the level of ROS generated by primary tumors [85]. Several studies have identified that the inhibition of mitochondrial ROS is an effective therapeutic strategy for control and prevention of metastasis [86,87]. On the contrary, melanoma cells circulating in the blood stream undergoes several reversible metabolic changes to withstand higher oxidative stress [88]. Here, antioxidants may quench the excessive ROS in the circulating blood that cause severe cellular damages and facilitate metastasis. Perhaps, this is one of the reasons where antioxidant therapy succeeds or fails to bring effective remission and recovery of malignant tumours. Therefore, it is necessary to device strategies to eliminate ROS from both mitochondrial and cellular compartments using antioxidant compounds.

## 5. Metabolic Effects of Antioxidants

The accumulation of ROS and Kreb’s cycle metabolites cause the constitutive activation of Hif-1 even under normoxic conditions. Under hypoxic conditions, the activation of Hif-1 is capable of inducing metabolic genes facilitating the tumor growth [89]. The term ’oncometabolites’ relates to the specific metabolites that arise in the neoplastic cells as a result of the altered or reprogrammed metabolic pathways. The common oncometabolites found in the cancer cells are 2-hydroxybutarate (2-HG), fumerate, maleate and succinate. They can assist in reprogramming metabolic pathways and change mitochondrial dynamics leading to pathologies. These include increase in EMT, metastasis of cancer cells, induction of dedifferentiation, changes in epigenetic, inflammatory, and paracrine signaling properties [90,91].

**Figure 2:**
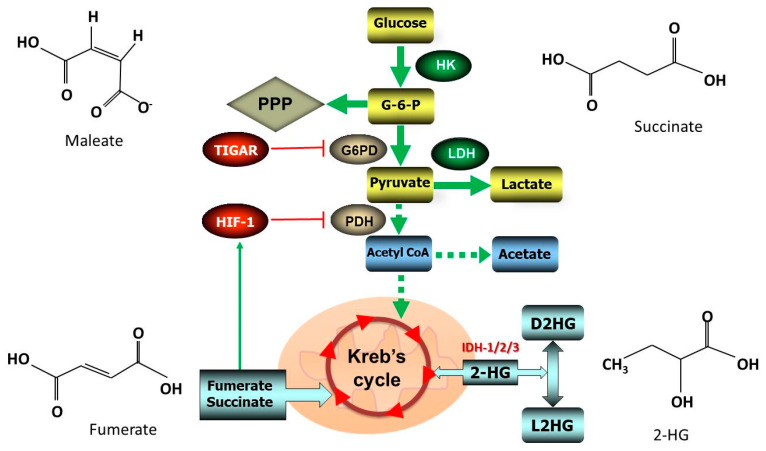
Metabolic regulation of antioxidants: Highly proliferative cancer cells rely on glycolysis to meet its exceedingly high energy demands. Activation of Hypoxia inducible factor-1 (Hif-1) cause the suppression of Pyruvate Dehydrogenase (PDH) resulting in the opening up of glucose transporters and increase in the flux of glucose in glycolytic cycle. During hypoxia, Hif-1 switches cell gene expressions from glycolysis to production and utilization of lactates (Warburg effect). The oncometabolites, fumerate and succinate may act on Hif-1 for inducing a ‘pseudohypoxic’ response. Besides, mutations in p53 attenuate TP53-induced Glycolysis and Apoptosis Regulator (TIGAR) causing a lack of inhibition on the Glucose-6-Phosphate Dehydrogenase (G6PD) enzyme activity leading to the increase in the metabolic flux through glycolytic and Pentose Phosphate Pathway (PPP). The D-2-hydroxyglutarate (D2HG) and the L-2-hydroxyglutarate (L2HG) are enantiomers of the metabolite 2-HG arising from the mutations in the Isocitrate Dehydrogenase 1/2/3 (IDH1/2/3). Eventually, these changes may cause altered metabolic flux responsible for the initiation of neoplasms. Thick solid lines indicate strong and dotted lines indicate weak flux of metabolites. Abbreviations: HK, Hexokinase; G-6-P, Glucose-6-Phosphate; LDH, Lactic Dehydrogenase.

Oncometabolites are generated due to the mutations in gene encoding Kreb’s cycle enzymes viz. isocitrate dehydrogenase 1, 2, and 3 (IDH1/2/3), fumarate hydratase, and succinate dehydrogenase. Among these three isoforms of IDH in human cells, the homodimeric IDH1/2 utilize NADP+ for the reversible conversion of isocitrate to α-ketoglutarate (α-KG), while IDH3 utilizes NAD+ for the irreversible conversion of the same metabolites [92]. The metabolite 2-HG exists as enantiomers D-2-hydroxyglutarate (D2HG) and the L-2-hydroxyglutarate (L2HG). Mutations in isocitrate dehydrogenase 1 or 2 (IDH1/2) results in the up rise of oncometabolite, D-2-hydroxyglutarate (D2HG), a reduced form of the α-ketoglutarate (Figure 2). This leads to the depletion of α-ketoglutarate in the cytoplasmic pool, which is a cosubstrate for the dioxygenases. Net result is the changes in epigenetics leading to the onset of neoplasms [93,94].

Oncometabolites are capable of inducing a ‘pseudohypoxic’ state for growing tumor mass due to the overexpression of mutant IDH1 and the elevated levels of Hif-1, as detected in the human cancer cell lines [95]. The increased expression of Hif-1 stimulates the activity of glucose transporters as well as hexokinase-2 resulting in the increased flux of glucose during glycolysis. However, the outcome of these changes on tumor mass and angiogenesis is variable with the enantiomers of 2-HG viz. D2HG and L2HG [96,97]. Similar to 2-HG, high levels of fumerate and succinate leaked into the cytoplasm are also capable of inducing Hif-1 and creating a ‘pseudohypoxic’ response [98]. An increase in fumerate may result in the inactivation of fumarate hydratase leading to oxidative stress due to depletion of NADPH and succination of glutathione leading to the formation of succinic-GSH (a covalent adduct of fumarate and glutathione) [99,100]. Moreover, succinate dehydrogenase will also alter the cellular redox potential facilitating tumorigenesis while participating in the transfer of electrons in the mitochondrial respiratory chain [101].

In healthy cells, p53 reduce glycolysis for the lowering of intracellular fructose-2,6-bisphosphate by increasing the expression of TIGAR. However, when p53 is mutated and TIGAR is attenuated, there will be a lack of inhibition on the Glucose-6-Phosphate dehydrogenase (G6PD) enzyme activity leading to the increase in the metabolic flux through glycolytic and PPP [60,102]. In fact, the catalysis of G6PD enzyme is the first irreversible step of the PPP for the generation of NADPH that is required for neutralizing intracellular oxidative stress. Thus, cancer cells are able to generate higher amount of NADP that helps them to tolerate higher oxidative stress during increased metabolic activity. Moreover, folate (an essential vitamin) metabolism, which involves the transfer of a carbon from serine hydroxymethyltransferase to tetrahydrofolate and methylene-tetrahydrofolate utilizes NAD+ and NADP+ for generating the reducing intermediates NADH as well as NADPH [103,104]. Perhaps, the use of target-specific antioxidant inhibitors against NADP/NADPH can elevate oxidative stress and attenuate tumor growth. Pre-clinical assays for understanding the side-effects of such antioxidant inhibitors have to be experimented in animal models.

Glutathione (GSH) is the most abundant antioxidant in living cells and is synthesized from glutamine-derived glutamate through condensation with the amino acids viz. cysteine (produced from extracellular cysteine) by glutamate-cysteine ligase and from glycine by glutathione synthase. In fact, maintenance of cysteine pool during glutamine oxidation supplies carbon to malic enzymes while producing NADPH for the cellular redox homeostasis. Among the various types of antioxidants, GSH play a major role in the protection of normal cells from excessive oxidative stress and maintenance of cellular redox balance. However, in neoplasms GSH has a decisive role in cell survival during hypoxia and nutrient deprivation, which is the rationale for the use of antioxidant inhibitors and GSH antagonists in anti-cancer therapy [22]. In proliferating cancer cells, GSH is responsible for (i) production of NADPH used for the biosynthesis of lipids and (ii) protection from oxidative stress by the reduction of oxidized GSH. However, an excess glutathione may promote tumor progression by detoxification of xenobiotics conferring therapeutic resistance and creating an environment for metastasis [105,106].

A high level of ROS is encountered in the cancer cells due to the loss of function of tumor suppressor genes along with the activation of oncogenes for signaling as well as metabolic pathways. Cancer cells make use of the excess glucose and oxygen availability to generate NADPH that helps to survive in the excessive oxidative stress environment. During hypoxic and low glucose conditions ROS will increase, which again will activate the antioxidant systems by altering the central carbon metabolism. Such conditions will also cause loss of matrix attachment leading to cell death or metastasis of tumor mass to distant locations [94]. Perhaps, antioxidants targeting mitochondria generated oxide free radicals and the inhibitors of oxidative stress enzymes can reduce the hypoxia induced activation of Hif-1. It is necessary to find the therapeutic relevance of these findings by estimating the proliferation and metastasis of tumor.

## 6. Antioxidants in Chemotherapy

Anti-cancer drugs are mostly cytotoxic and are associated with a wide range of side effects due to the alteration of the cellular oxidative status. Chemotherapeutic drugs often damage DNA by production of free radicals that interfere with cell cycle leading to cell death by apoptosis or necrosis [107]. Most of these chemotherapeutic agents act on fast dividing cells viz. bone marrow, epithelial cells etc. and a variety of tissues and organs viz. cardiomyocytes, hepatocytes, lungs, and kidneys. They may damage different organs and cause systemic problems while interfering with rapidly dividing cells. A possible solution would be the use of antioxidants along with the cancer chemotherapy that increase cellular oxidative stress. Thus, both antioxidants and prooxidants have clear advantages and disadvantages in cancer therapy (Table 2).

In general, the mode of action of anti-cancer agents involves increasing the oxidative stress leading to the interference of DNA replication and proliferation of the neoplastic cells. However, incorporation of antioxidants may reduce the efficacy of the anti-cancer therapy by interfering with the production of free radicals. Thus, many clinicians are reluctant to use the antioxidants during anti-neoplastic chemotherapeutic regimen. However, there are situations where antioxidants can work in synergy with many of the anti-cancer drug. Quantitatively, these antioxidants will allow higher and longer duration of intake of anti-neoplastic agents thereby increasing the effectiveness of therapy. A meta-analysis conducted on the use of antioxidants alongside the cancer chemotherapy indicates that they increase the therapeutic potential as well as survival times in cancer patients [6].

Anti-cancer antioxidant therapies can be divided into two categories as any other types of medical supplementations (1) prophylactic dose, which is a lower dose rendering protection to normal cells and tumor cells and (2) therapeutic dose, which is a higher dose inhibiting the growth of cancer cells without interfering with normal cell growth. It is quite difficult to make meaningful comparisons due to the variations in the cancer types, study designs, therapeutic regimen, and statistical analysis between the studies. However, there are enough evidences to conclude that antioxidants enhance cytotoxic effect of chemotherapy by promoting therapeutic benefits of anti-cancer therapy and an increase in patient survival.

Chemotherapy increases oxidative stress, which is instrumental for the killing of cancer cells, although, a higher level of oxidative stress may often result in the ineffectiveness of some anti-cancer drugs. During higher oxidative stress conditions cancer cell replicates slowly, which may decrease the efficacy of chemotherapy [117]. Alternatively, free radicals can increase oxidation of cellular proteins into carbonyls resulting in the inhibition of caspases and the consequent inhibition of apoptosis reduces the efficacy of cancer chemotherapy. Antioxidants may reduce the formation of carbonyls viz. aldehydes and ketones, which allows the functioning of caspases and enhanced efficacy of anti-cancer drugs [7]. Antioxidants may also increase cytotoxic effects of anti-neoplastic drugs to undergo apoptosis instead of necrosis. Concomitantly, they can reduce cytotoxicity arising from the action anti-cancer drug on normal cells.

The beneficial effect of antioxidants may be attributed to its capacity to lower the side-effects from excessive free radical formation, while maintaining the necessary cellular oxidative stress during anti-cancer chemotherapeutic regimen. In effect, chemotherapy reduces the serum levels of antioxidant vitamins and minerals due to lipid peroxidation resulting in higher levels of oxidative stress. The supplementation of antioxidants and vitamin C and E was found to reduce the risk of recurrence of breast cancer [118]. Vitamin C makes its entry into cells through glucose transporters to exert antioxidant effects, thereby keeping the oxidative stress low. Vitamin E has the additional benefit of decreasing chemotherapy mediated toxicity by suppressing lipid peroxidation and inducing apoptosis of tumor cells [119].

## 7. Role of Photodynamic Therapy

Mitochondrial energy metabolism is the primary source of oxide free radicals. Free radicals such as hydrogen peroxide (H_2_O_2_) and superoxide (^•^O_2_) radicals increases the rate of cellular DNA replication and proliferation. An excess number of free radicals may result in harmful consequences leading to systemic diseases and cancer. Any imbalances in the cellular redox status either by an increase in free radicals or an escalation of reducing agents, favors growth arrest and apoptosis [120]. In fact, the mechanism of action of non-ionizing and ionizing radiations in killing cancer cells involves the generation of abnormally high levels of free radicals in the body. Photodynamic therapy (PDT) using non-ionizing radiation engages light-sensitive compound (photosensitizer) to react with molecular oxygen (O_2_) for generating excess free radicals, especially hydrogen peroxide (H_2_O_2_) and superoxide (^•^O_2_) radicals to evoke growth arrest and cell death. On the contrary, radiation therapy with ionizing radiations result in the formation of oxygen radicals in the living cells by interaction of radiation with water, a process known as radiolysis [121].

A combination of synthetic and natural antioxidants serves as radioprotectants for patients on radiation therapy. Antioxidants such as vitamin E (α-tocopherol), melatonin and retinol palmitate has been effective against radiation-induced mucositis, brain cancer and radiation-induced proctopathy respectively [122]. Instead, PDT can serve as therapeutic modality for inducing oxide free radicals that can stimulate cell signaling leading to antitumor immune response, reduction of tumour mass, apoptosis, and necrosis [123]. Unlike the action of ionizing radiations on biological systems resulting in the formation of excessive amounts of oxide radicals by untargeted radiations, PDT cause a controlled production of oxidative stress by using specific drug(s) and targeted response. However, the presence of antioxidants in photodynamic reactions reduces the efficacy of PDT, although, a few of them viz. ascorbic acid, α-tocopherol or butyl-4-hydroxyanisole may possibly enhance the photodynamic effect in malignant cells [124]. And, the exact reasons behind the variable nature of these antioxidants on cancers need closer investigations.

PDT involves in the production of free radicals leading to the oxidation of proteins and a consequent remission of angiogenesis in tumour mass. Although, free radicals are short-lived and survive only up to microseconds, their secondary metabolites bring out changes in the redox status as well as biophysiological activities leading to the cellular activities viz. proliferation, differentiation, migration etc. An increase in hypoxia and ROS results in the stimulation of the signaling pathways involving Ask1-induced immediate early stress response followed by the activation of Hif-1. Oxidative stress induced by PDT is also instrumental for the onset of survival mechanisms by the tumor mass viz. activation of transcriptional factors (AP-1, Hif-1, NF*k*B, Nrf2 etc.) leading to antioxidant response. Moreover, PDT is also responsible for the oxidation of proteins by activating transcription factor (Atf) 4 and 6, heat shock factor-1 (Hsf-1), and X-box binding protein 1 (Xbp1) [123]. In general, cells undergone PDT may follow survival mechanisms by activating various cellular stress responses and restoring intracellular redox status [125].

An excessive oxidative stress is characterized by acute cellular death, while sub-lethal levels of oxidative stress leads to up regulation of antioxidants, cellular efflux mechanisms and detoxifying enzymes. Till date, no specific studies have been conducted to analyze the effect of PDT on various antioxidant enzymes and transcription factors. It is speculated that PDT increase ROS by activating the Ask1 pathway leading to the transmission of signal through JNK1 and p38 MAPK to phosphorylate Nrf2 at Ser40. This results in the translocation of Nrf2 to the nucleus and dimerization with AP-1 transcription factors leading to the expression of GSH and various multidrug transporters [126]. PDT induces the expression of several genes involved in the angiogenesis, inflammation, proliferation, and survival by activation of NF*k*B [127]. Hypoxia induced by PDT results in the suppression of angiogenesis as well as reduction in the enzymatic activity of prolyl hydroxylase domain enzymes and the factor inhibiting Hif. This results in the stabilization of Hif-1 for nuclear translocation, dimerization, and binding of ARE sequences. Ultimately, this leads to the upregulation of genes involved in the survival mechanisms, angiogenesis and proliferation or apoptosis and tumor regression [123].

In summary, PDT can modulate cellular antioxidant responses through Nrf2, proinflammatory response through NF*k*B and survival response during oxidative stress through Hif-1. Additionally, PDT is responsible for stress response mediated by Ask1, JNK, MAPK and a wide range of transcription factors. Thus, blocking of these signaling modulators or their downstream protein products cycloxygenase-2, heme oxygenase-1, or heat shock protein-70 may alter the levels of free radicals and improve the efficiency of PDT. Nanomedicine-aided PDT has greater specificity for the destruction of cancer cells with minimal side-effects. Photosensitizers along with target-specific molecules can also accumulate in the lipid bilayer of cancer stem cells to generate free radicals upon irradiation with light of suitable wavelength leading to the remission of tumor mass. Indeed, targeted PDT along with suitable adjuvants and immunostimulants will be another feasible option to eradicate such recalcitrant tumors [128].

## 8. Conclusions

Antioxidants are molecules often referred to as ’scavengers of free radicals’ that are capable of interacting with free radicals and neutralizing them in the living systems. It can be synthesized either by the body (endogenous antioxidants) or obtained from the external (exogenous) sources, mainly from the diet. Although, several pre-clinical and clinical studies have shown mixed results, researchers believe that the exogenous antioxidants (especially dietary antioxidants) can quench the excess levels of free radicals, which may be useful to reduce cellular damages associated with cancer chemotherapy and radiotherapy [129]. Chemotherapy and radiotherapy increase the oxidative and nitrosative radicals that can lead to increase in cytotoxicity for anti-cancer effects. Use of antioxidants during anti-cancer therapy may help to protect both diseased as well as healthy cells, thereby compromising the efficacy of treatment. Thus, many clinicians are wary on prescribing antioxidants, especially vitamins and supplements during intense chemo/radio therapies. Research has found that several antioxidants work selectively on the diseased as well as normal cells, based on their biodistribution as well as mechanism of action. There are also observations that the antioxidants reduce the cytotoxic effects of the anticancer agents on the healthy cells. It is noteworthy that the supplementation of vitamin C is found to protect healthy cells against the damages caused by excessive oxidative stress and the risk for developing cancer due to cigarette smoking [130]. Moreover, both clinicians and researchers believe that the concurrent use of antioxidants remove the excessive levels of free radicals and create a congenial environment for healthy cell and tissue repair. Perhaps, it is more suitable to stick to selected antioxidants as dietary supplementation during the course of the disease.

## Figures and Tables

**Table 1:** Mutations in the *Nrf-KeapI* system responsible for cancer [69].

Gene	Somatic Mutations	Cancer Types	References
*KeapI*	C23Y, D256G	Breast cancer	[70,71]
	R234W, T330I, V660M,	Colorectal adenocarcinoma	[70,72]
	R470C	Cervical squamous carcinoma	[73]
	F107L, A159T, A188V, P412S, E611K	Epithelial ovarian cancer	[74]
	Q82H, S233N, F280L	Gastric adenocarcinoma	[70]
	S338L, G379D,	Gall bladder adenocarcinoma	[75]
	N183S, D236Y, L342M	Hepatocellular carcinoma	[70]
	C249Y, W554X	Liver cholangiocarcinoma	[75]
	E244K, G364C, R415G, G430C, W591L	Lung adenocarcinoma	[70,76]
	G333C	Lung non-small-cell carcinoma	[77]
	V42A, S45F, R320Q	Lung squamous cell carcinoma	[70,78]
	Y255H, T314M, D357N,	Prostrate adenocarcinoma	[70,75]
*Nrf2*	W24C, D29Y, R34Q, D77A, G81D	Cervical squamous carcinoma	[73,79]
	D27Y, E78K, E79K, T80K, G81V, E82L	Esophagus squamous carcinoma	[79,80]
	D29G, Q75H, T80I	Head and neck cancer	[75]
	D29H, D77N, E79G	Larynx carcinoma	[80]
	E79Q,	Lung adenocarcinoma	[75]
	D77V, E79K, R34L, R34Q, R34Q	Lung non-small-cell carcinoma	[75,81]
	D77A, E79K, T80R, G81D, E82G	Lung squamous cell carcinoma	[75,76,80]
	L30F	Malignant melanoma	[79]
	G31A	Skin squamous cell carcinoma	[80]

**Table 2:** Advantages and disadvantages of anti- and pro-oxidant therapies in cancer.

	Advantages	Disadvantages	References
Antioxidants	Antagonize excess free radicals in healthy cells	Cancers may also benefit from the use of antioxidants	[13,45,58]
	Enhancement of host antitumor immunity	Antioxidants might prevent cancers than curing them	[47,108]
	Available in a wide variety of fruits and vegetables	Difficulty in delivery to tumor mass or internal organs	[109,110]
	Short-lived and ultra-fast acting oxidizing biomolecules	Acts on DNA causing mutations and increasing cancer	[111,112,113]
Prooxidants	Increase oxidative stress hindering cancer growth	Damage healthy tissues, especially the internal organs	[23,114]
	Synergistic action with many anti-cancer drugs	Increase cytotoxicity during radiotherapy	[115,116]

## References

[1] Murphy M.P. (2009). How mitochondria produce reactive oxygen species. Biochem. J..

[2] Maiolino G., Rossitto G., Caielli P., Bisogni V., Rossi G.P., Calò L.A. (2013). The Role of Oxidized Low-Density Lipoproteins in Atherosclerosis: The Myths and the Facts. Mediat. Inflamm..

[3] Tinkel J., Hassanain H., Khouri S.J. (2012). Cardiovascular Antioxidant Therapy. Cardiol. Rev..

[4] Song Y., Cook N.R., Albert C.M., Van Denburgh M., Manson J.E. (2009). Effects of vitamins C and E and β-carotene on the risk of type 2 diabetes in women at high risk of cardiovascular disease: A randomized controlled trial. Am. J. Clin. Nutr..

[5] Kunwar A., Priyadarsini K.I. (2011). Free radicals, oxidative stress and importance of antioxidants in human health. J. Med. Allied Sci..

[6] Singh K., Bhori M., Kasu Y.A., Bhat G., Marar T. (2018). Antioxidants as precision weapons in war against cancer chemotherapy induced toxicity—Exploring the armoury of obscurity. Saudi Pharm. J..

[7] Conklin K.A. (2004). Chemotherapy-Associated Oxidative Stress: Impact on Chemotherapeutic Effectiveness. Integr. Cancer Ther..

[8] Beckman K.B., Ames B.N. (1997). Oxidative Decay of DNA. J. Biol. Chem..

[9] Sarangarajan R., Meera S., Rukkumani R., Sankar P., Anuradha G. (2017). Antioxidants: Friend or foe?. Asian Pac. J. Trop. Med..

[10] Bergendi L., Beneš L., Ďuračková Z., Ferenčik M. (1999). Chemistry, physiology and pathology of free radicals. Life Sci..

[11] George S., Hamblin M.R., Abrahamse H. (2019). Differentiation of Mesenchymal Stem Cells to Neuroglia: In the Context of Cell Signalling. Stem Cell Rev. Rep..

[12] Lee S., Hashimoto J., Suzuki T., Satoh A. (2017). The effects of exercise load during development on oxidative stress levels and antioxidant potential in adulthood. Free. Radic. Res..

[13] Valko M., Leibfritz D., Moncol J., Cronin M.T., Mazur M., Telser J. (2007). Free radicals and antioxidants in normal physiological functions and human disease. Int. J. Biochem. Cell Biol..

[14] Pham-Huy L.A., He H., Pham-Huy C. (2008). Free Radicals, Antioxidants in Disease and Health. Int. J. Biomed. Sci..

[15] Nimse S.B., Pal D. (2015). Free radicals, natural antioxidants, and their reaction mechanisms. RSC Adv..

[16] Ighodaro O., Akinloye O. (2018). First line defence antioxidants-superoxide dismutase (SOD), catalase (CAT) and glutathione peroxidase (GPX): Their fundamental role in the entire antioxidant defence grid. Alex. J. Med..

[17] Chelikani P., Fita I., Loewen P.C. (2004). Diversity of structures and properties among catalases. Cell. Mol. Life Sci..

[18] Radi R., Turrens J.F., Chang L.Y., Bush K.M., Crapo J.D., Freeman B.A. (1991). Detection of catalase in rat heart mitochondria. J. Biol. Chem..

[19] Marengo B., Nitti M., Furfaro A.L., Colla R., De Ciucis C., Marinari U.M., Pronzato M.A., Traverso N., Domenicotti C. (2016). Redox Homeostasis and Cellular Antioxidant Systems: Crucial Players in Cancer Growth and Therapy. Oxidative Med. Cell. Longev..

[20] Sies H. (1999). Glutathione and its role in cellular functions. Free. Radic. Biol. Med..

[21] Birk J., Meyer M., Aller I., Hansen H.G., Odermatt A., Dick T.P., Meyer A.J., Appenzeller-Herzog C. (2013). Endoplasmic reticulum: Reduced and oxidized glutathione revisited. J. Cell Sci..

[22] Traverso N., Ricciarelli R., Nitti M., Marengo B., Furfaro A.L., Pronzato M.A., Marinari U.M., Domenicotti C. (2013). Role of Glutathione in Cancer Progression and Chemoresistance. Oxidative Med. Cell. Longev..

[23] Li B., Cao Y., Meng G., Qian L., Xu T., Yan C., Luo O., Wang S., Wei J., Ding Y. (2019). Targeting glutaminase 1 attenuates stemness properties in hepatocellular carcinoma by increasing reactive oxygen species and suppressing Wnt/beta-catenin pathway. EBio. Med..

[24] Murphy M.P. (2012). Mitochondrial Thiols in Antioxidant Protection and Redox Signaling: Distinct Roles for Glutathionylation and Other Thiol Modifications. Antiox. Redox. Signal..

[25] Molavian H., Goldman A., Phipps C.J., Kohandel M., Wouters B.G., Sengupta S., Sivaloganathan S. (2016). Drug-induced reactive oxygen species (ROS) rely on cell membrane properties to exert anticancer effects. Sci. Rep..

[26] Lubos E., Loscalzo J., Handy D.E. (2011). Glutathione Peroxidase-1 in Health and Disease: From Molecular Mechanisms to Therapeutic Opportunities. Antiox. Redox. Signal..

[27] Ryter S.W., Choi A.M.K. (2005). Heme Oxygenase-1: Redox Regulation of a Stress Protein in Lung and Cell Culture Models. Antiox. Redox. Signal..

[28] Kim J.-S., Roberts J.M., Bingman W.E., Shao L., Wang J., Ittmann M.M., Weigel N. (2014). The Prostate Cancer TMPRSS2:ERG Fusion Synergizes With the Vitamin D Receptor (VDR) to Induce CYP24A1 Expression-Limiting VDR Signaling. Endocrinology.

[29] Cox A.G., Winterbourn C.C., Hampton M.B. (2009). Mitochondrial peroxiredoxin involvement in antioxidant defence and redox signalling. Biochem. J..

[30] Cao J., Schulte J., Knight A., Leslie N.R., Zagozdzon A., Bronson R., Manevich Y., Beeson C., Neumann C.A. (2009). Prdx1 inhibits tumorigenesis via regulating PTEN/AKT activity. EMBO J..

[31] Fridovich I. (1995). Superoxide Radical and Superoxide Dismutases. Annu. Rev. Biochem..

[32] Sheng Y., Abreu I.A., Cabelli D.E., Maroney M.J., Miller A.-F., Teixeira M., Valentine J.S. (2014). Superoxide Dismutases and Superoxide Reductases. Chem. Rev..

[33] Lebovitz R.M., Zhang H., Vogel H., Cartwright J., Dionne L., Lu N., Huang S., Matzuk M.M. (1996). Neurodegeneration, myocardial injury, and perinatal death in mitochondrial superoxide dismutase-deficient mice. Proc. Natl. Acad. Sci. USA.

[34] Roberts B.R., Tainer J.A., Getzoff E.D., Malencik D.A., Anderson S.R., Bomben V.C., Meyers K.R., Karplus P.A., Beckman J.S. (2007). Structural Characterization of Zinc-deficient Human Superoxide Dismutase and Implications for ALS. J. Mol. Biol..

[35] Dayal S., Baumbach G.L., Arning E., Bottiglieri T., Faraci F.M., Lentz S.R. (2017). Deficiency of superoxide dismutase promotes cerebral vascular hypertrophy and vascular dysfunction in hyperhomocysteinemia. PLoS ONE.

[36] Lincoln D.T., Emadi E.M.A., Tonissen K.F., Clarke F.M. (2003). The thioredoxin-thioredoxin reductase system: Over-expression in human cancer. Anticancer. Res..

[37] Saitoh M., Nishitoh H., Fujii M., Takeda K., Tobiume K., Sawada Y., Kawabata M., Miyazono K., Ichijo H. (1998). Mammalian thioredoxin is a direct inhibitor of apoptosis signal-regulating kinase (ASK) 1. EMBO J..

[38] Harris I.S., Treloar A.E., Inoue S., Sasaki M., Gorrini C., Lee K.C., Young K.Y., Brenner D., Knobbe-Thomsen C.B., Cox M. (2015). Glutathione and thioredoxin antioxidant pathways synergize to drive cancer initiation and progression. Cancer Cell.

[39] Fiedor J., Burda K. (2014). Potential Role of Carotenoids as Antioxidants in Human Health and Disease. Nutrients.

[40] Kurutas E.B. (2015). The importance of antioxidants which play the role in cellular response against oxidative/nitrosative stress: Current state. Nutr. J..

[41] Niki E. (2014). Role of vitamin E as a lipid-soluble peroxyl radical scavenger: In vitro and in vivo evidence. Free. Radic. Biol. Med..

[42] Rice-Evans C.A., Miller N.J., Paganga G. (1996). Structure-antioxidant activity relationships of flavonoids and phenolic acids. Free. Radic. Biol. Med..

[43] Yu G.-M., Kubota H., Okita M., Maeda T. (2017). The anti-inflammatory and antioxidant effects of melatonin on LPS-stimulated bovine mammary epithelial cells. PLoS ONE.

[44] Oikawa S., Yamada K., Yamashita N., Tada-Oikawa S., Kawanishi S. (1999). N-acetylcysteine, a cancer chemopreventive agent, causes oxidative damage to cellular and isolated DNA. Carcinogenesis.

[45] Harris I.S., DeNicola G.M. (2020). The Complex Interplay between Antioxidants and ROS in Cancer. Trends Cell Biol..

[46] Sagun K.C., Càrcamo J.M., Golde D.W. (2005). Vitamin C enters mitochondria via facilitative glucose transporter 1 (Gluti) and confers mitochondrial protection against oxidative injury. FASEB J..

[47] Chen Q., Espey M.G., Sun A.Y., Pooput C., Kirk K.L., Krishna M.C., Khosh D.B., Drisko J., Levine M. (2008). Pharmacologic doses of ascorbate act as a prooxidant and decrease growth of aggressive tumor xenografts in mice. Proc. Natl. Acad. Sci. USA.

[48] Wilson J.X. (2005). Regulation of Vitamin C Transport. Annu. Rev. Nutr..

[49] Wagner E.F., Nebreda Á.R. (2009). Signal integration by JNK and p38 MAPK pathways in cancer development. Nat. Rev. Cancer.

[50] Yuan T.L., Cantley L.C. (2008). PI3K pathway alterations in cancer: Variations on a theme. Oncogene.

[51] Ahn C.S., Metallo C.M. (2015). Mitochondria as biosynthetic factories for cancer proliferation. Cancer Metab..

[52] Myant K.B., Cammareri P., McGhee E.J., Ridgway R.A., Huels D.J., Cordero J.B., Schwitalla S., Kalna G., Ogg E.-L., Athineos D. (2013). ROS production and NF-kappaB activation triggered by RAC1 facilitate WNT-driven intestinal stem cell proliferation and colorectal cancer initiation. Cell. Stem Cell..

[53] Wang P., Wu J., Ma S., Zhang L., Yao J., Hoadley K.A., Wilkerson M.D., Perou C.M., Guan K.-L., Ye D. (2015). Oncometabolite D-2-Hydroxyglutarate Inhibits ALKBH DNA Repair Enzymes and Sensitizes IDH Mutant Cells to Alkylating Agents. Cell Rep..

[54] Antunes F., Han D. (2009). Redox regulation of NF-kappaB: From basic to clinical research. Antiox. Redox. Signal.

[55] Morgan M.J., Liu Z.-G. (2010). Crosstalk of reactive oxygen species and NF-κB signaling. Cell Res..

[56] Hussain S.P., Amstad P., He P., Robles A., Lupold S., Kaneko I., Ichimiya M., Sengupta S., Mechanic L., Okamura S. (2004). p53-Induced Up-Regulation of MnSOD and GPx but not Catalase Increases Oxidative Stress and Apoptosis. Cancer Res..

[57] Nam S.Y., Sabapathy K. (2011). p53 promotes cellular survival in a context-dependent manner by directly inducing the expression of haeme-oxygenase-1. Oncogene.

[58] Sayin V.I., Ibrahim M.X., Larsson E., Nilsson J.A., Lindahl P., Bergo M.O. (2014). Antioxidants Accelerate Lung Cancer Progression in Mice. Sci. Transl. Med..

[59] Polyak K., Xia Y., Zweier J.L., Kinzler K.W., Vogelstein B. (1997). A model for p53-induced apoptosis. Nat. Cell Biol..

[60] Bensaad K., Tsuruta A., Selak M.A., Vidal M.N.C., Nakano K., Bartrons R., Gottlieb E., Vousden K.H. (2006). TIGAR, a p53-Inducible Regulator of Glycolysis and Apoptosis. Cell.

[61] Hu M., Liu L., Yao W. (2018). Activation of p53 by costunolide blocks glutaminolysis and inhibits proliferation in human colorectal cancer cells. Gene.

[62] Sablina A.A., Budanov A.V., Ilyinskaya G.V., Agapova L.S., Kravchenko J.E., Chumakov P.M. (2005). The antioxidant function of the p53 tumor suppressor. Nat. Med..

[63] Kruiswijk F., Labuschagne C.F., Vousden K.H. (2015). p53 in survival, death and metabolic health: A lifeguard with a licence to kill. Nat. Rev. Mol. Cell Biol..

[64] Jaramillo M.C., Zhang D.D. (2013). The emerging role of the Nrf2-Keap1 signaling pathway in cancer. Genes Dev..

[65] Cebula M., Schmidt E.E., Arnér E.S.J. (2015). TrxR1 as a Potent Regulator of the Nrf2-Keap1 Response System. Antiox. Redox Signal..

[66] DeNicola G.M., Chen P.-H., Mullarky E., Sudderth J.A., Hu Z., Wu D., Tang H., Xie Y., Asara J.M., Huffman K.E. (2015). NRF2 regulates serine biosynthesis in non–small cell lung cancer. Nat. Genet..

[67] Harvey C., Thimmulappa R., Singh A., Blake D., Ling G., Wakabayashi N., Fujii J., Myers A., Biswal S. (2009). Nrf2-regulated glutathione recycling independent of biosynthesis is critical for cell survival during oxidative stress. Free. Radic. Biol. Med..

[68] Wakabayashi N., Slocum S.L., Skoko J.J., Shin S., Kensler T.W. (2010). When NRF2 talks, who’s listening?. Antiox. Redox Signal..

[69] Sova M., Saso L. (2018). Design and development of Nrf2 modulators for cancer chemoprevention and therapy: A review. Drug Des. Dev. Ther..

[70] Yoo N.J., Kim H.R., Kim Y.R., An C.H., Lee S.H. (2012). Somatic mutations of the KEAP1 gene in common solid cancers. Histopathology.

[71] Nioi P., Nguyen T. (2007). A mutation of Keap1 found in breast cancer impairs its ability to repress Nrf2 activity. Biochem. Biophys. Res. Commun..

[72] Zhang P., Singh A., Yegnasubramanian S., Esopi D., Kombairaju P., Bodas M., Wu H., Bova G.S., Biswal S. (2010). Loss of Keap1 function in prostate cancer cells causes chemo- and radio-resistance and promotes tumor growth. Mol. Cancer Ther..

[73] Chu X.Y., Li Z.J., Zheng Z.W., Tao Y.L., Zou F.X., Yang X.F. (2018). KEAP1/NRF2 signaling pathway mutations in cervical cancer. Eur. Rev. Med. Pharmacol. Sci..

[74] Konstantinopoulos P.A., Spentzos D., Fountzilas E., Francoeur N., Sanisetty S., Grammatikos A.P., Hecht J.L., Cannistra S.A. (2011). Keap1 Mutations and Nrf2 Pathway Activation in Epithelial Ovarian Cancer. Cancer Res..

[75] Shibata T., Kokubu A., Gotoh M., Ojima H., Ohta T., Yamamoto M., Hirohashi S. (2008). Genetic Alteration of Keap1 Confers Constitutive Nrf2 Activation and Resistance to Chemotherapy in Gallbladder Cancer. Gastroenterology.

[76] Padmanabhan B., Tong K.I., Ohta T., Nakamura Y., Scharlock M., Ohtsuji M., Kang M.-I., Kobayashi A., Yokoyama S., Yamamoto M. (2006). Structural Basis for Defects of Keap1 Activity Provoked by Its Point Mutations in Lung Cancer. Mol. Cell.

[77] Singh A., Misra V., Thimmulappa R.K., Lee H., Ames S., Hoque M.O., Herman J.G., Baylin S.B., Sidransky D., Gabrielson E. (2006). Dysfunctional KEAP1–NRF2 interaction in non-small-cell lung cancer. PLoS Med..

[78] Gong M., Li Y., Ye X., Zhang L., Wang Z., Xu X., Shen X., Zheng C. Loss-of-Function Mutations in KEAP1 Drive Lung Cancer Progression via KEAP1/NRF2 Pathway Activation. https://assets.researchsquare.com/files/rs-8944/v2/manuscript.pdf.

[79] Shibata T., Kokubu A., Saito S., Narisawa-Saito M., Sasaki H., Aoyagi K., Yoshimatsu Y., Tachimori Y., Kushima R., Kiyono T. (2011). NRF2 Mutation Confers Malignant Potential and Resistance to Chemoradiation Therapy in Advanced Esophageal Squamous Cancer. Neoplasia.

[80] Kim Y.R., Oh J.E., Kim M.S., Kang M.R., Park S.W., Han J.Y., Eom H.S., Yoo N.J., Lee S.H. (2009). Oncogenic NRF2 mutations in squamous cell carcinomas of oesophagus and skin. J. Pathol..

[81] Kerins M.J., Ooi A. (2018). A catalogue of somatic NRF2 gain-of-function mutations in cancer. Sci. Rep..

[82] Antal C.E., Hudson A.M., Kang E., Zanca C., Wirth C., Stephenson N.L., Trotter E.W., Gallegos L.L., Miller C.J., Furnari F.B. (2015). Cancer-Associated Protein Kinase C Mutations Reveal Kinase’s Role as Tumor Suppressor. Cell.

[83] Leslie N.R. (2006). The Redox Regulation of PI 3-Kinase–Dependent Signaling. Antioxidants Redox Signal..

[84] Chang L., Graham P.H., Hao J., Ni J., Bucci J., Cozzi P.J., Kearsley J.H., Li Y. (2013). Acquisition of epithelial–mesenchymal transition and cancer stem cell phenotypes is associated with activation of the PI3K/Akt/mTOR pathway in prostate cancer radioresistance. Cell Death Dis..

[85] Gorrini C., Harris I.S., Mak T.W. (2013). Modulation of oxidative stress as an anticancer strategy. Nat. Rev. Drug Discov..

[86] Goh J., Enns L.C., Fatemie S., Hopkins H., Morton J., Pettan-Brewer C., Ladiges W.C. (2011). Mitochondrial targeted catalase suppresses invasive breast cancer in mice. BMC Cancer.

[87] Porporato P.E., Payen V.L., Pérez-Escuredo J., De Saedeleer C.J., Danhier P., Copetti T., Dhup S., Tardy M., Vazeille T., Bouzin C. (2014). A Mitochondrial Switch Promotes Tumor Metastasis. Cell Rep..

[88] Piskounova E., Agathocleous M., Murphy M.M., Hu Z., Huddlestun S.E., Zhao Z., Leitch A.M., Johnson T.M., DeBerardinis R.J., Morrison S.J. (2015). Oxidative stress inhibits distant metastasis by human melanoma cells. Nat. Cell Biol..

[89] Intlekofer A.M., DeMatteo R.G., Venneti S., Finley L.W.S., Lu C., Judkins A.R., Rustenburg A.S., Grinaway P.B., Chodera J.D., Cross J.R. (2015). Hypoxia Induces Production of L-2-Hydroxyglutarate. Cell Metab..

[90] Corrado M., Scorrano L., Campello S. (2016). Changing perspective on oncometabolites: From metabolic signature of cancer to tumorigenic and immunosuppressive agents. Oncotarget.

[91] Colvin H., Nishida N., Konno M., Haraguchi N., Takahashi H., Nishimura J., Hata T., Kawamoto K., Asai A., Tsunekuni K. (2016). Oncometabolite D-2-Hydroxyglurate Directly Induces Epithelial-Mesenchymal Transition and is Associated with Distant Metastasis in Colorectal Cancer. Sci. Rep..

[92] Frezza C., Pollard P.J., Gottlieb E. (2011). Inborn and acquired metabolic defects in cancer. J. Mol. Med..

[93] Xu W., Yang H., Liu Y., Yang Y., Wang P., Kim S.-H., Ito S., Yang C., Wang P., Xiao M.-T. (2011). Oncometabolite 2-Hydroxyglutarate Is a Competitive Inhibitor of α-Ketoglutarate-Dependent Dioxygenases. Cancer Cell.

[94] DeBerardinis R.J., Chandel N.S. (2016). Fundamentals of cancer metabolism. Sci. Adv..

[95] Zhao S., Lin Y., Xu W., Jiang W., Zha Z., Wang P., Yu W., Li Z., Gong L., Peng Y. (2009). Glioma-Derived Mutations in IDH1 Dominantly Inhibit IDH1 Catalytic Activity and Induce HIF-1. Science.

[96] Koivunen P., Lee S., Duncan C.G., Lopez G.Y., Lu G., Ramkissoon S.H., Losman J.A., Joensuu P., Bergmann U., Gross S. (2012). Transformation by the (R)-enantiomer of 2-hydroxyglutarate linked to EGLN activation. Nat. Cell Biol..

[97] Wang G.L., Jiang B.H., Rue E.A., Semenza G.L. (1995). Hypoxia-inducible factor 1 is a basic-helix-loop-helix-PAS heterodimer regulated by cellular O2 tension. Proc. Natl. Acad. Sci. USA.

[98] Selak M.A., Armour S.M., MacKenzie E.D., Boulahbel H., Watson D.G., Mansfield K.D., Pan Y., Simon M., Thompson C.B., Gottlieb E. (2005). Succinate links TCA cycle dysfunction to oncogenesis by inhibiting HIF-α prolyl hydroxylase. Cancer Cell.

[99] Sullivan L.B., Martinez-Garcia E., Nguyen H., Mullen A.R., Dufour E., Sudarshan S., Licht J.D., DeBerardinis R.J., Chandel N.S. (2013). The Proto-oncometabolite Fumarate Binds Glutathione to Amplify ROS-Dependent Signaling. Mol. Cell.

[100] Zheng L., Cardaci S., Jerby L., MacKenzie E.D., Sciacovelli M., Johnson T.I., Gaude E., King A., Leach J.D.G., Edrada-Ebel R. (2015). Fumarate induces redox-dependent senescence by modifying glutathione metabolism. Nat. Commun..

[101] Miettinen M., Lasota J. (2014). Succinate dehydrogenase deficient gastrointestinal stromal tumors (GISTs)—A review. Int. J. Biochem. Cell Biol..

[102] Jiang P., Du W., Wang X., Mancuso A.A., Gao X., Wu M., Yang X. (2011). p53 regulates biosynthesis through direct inactivation of glucose-6-phosphate dehydrogenase. Nat. Cell Biol..

[103] Nilsson R., Jain M., Madhusudhan N., Sheppard N.G., Strittmatter L., Kampf C., Huang J., Asplund A., Mootha V.K. (2014). Metabolic enzyme expression highlights a key role for MTHFD2 and the mitochondrial folate pathway in cancer. Nat. Commun..

[104] Ye J., Fan J., Venneti S., Wan Y.-W., Pawel B.R., Zhang J., Finley L.W., Lu C., Lindsten T., Cross J.R. (2014). Serine Catabolism Regulates Mitochondrial Redox Control during Hypoxia. Cancer Discov..

[105] Nguyen T.-L., Durán R.V. (2018). Glutamine metabolism in cancer therapy. Cancer Drug Resist..

[106] Bansal A., Simon M.C. (2018). Glutathione metabolism in cancer progression and treatment resistance. J. Cell Biol..

[107] Lamson D.W., Brignall M.S. (1999). Antioxidants in cancer therapy; their actions and interactions with oncologic therapies. Altern. Med. Rev. J. Clin. Ther..

[108] Hennekens C.H., Buring J.E., Manson J.E., Stampfer M., Rosner B., Cook N.R., Belanger C., LaMotte F., Gaziano J.M., Ridker P.M. (1996). Lack of Effect of Long-Term Supplementation with Beta Carotene on the Incidence of Malignant Neoplasms and Cardiovascular Disease. N. Engl. J. Med..

[109] Pantuck A.J., Leppert J.T., Zomorodian N., Aronson W., Hong J., Barnard R.J., Seeram N., Liker H., Wang H., Elashoff R. (2006). Phase II Study of Pomegranate Juice for Men with Rising Prostate-Specific Antigen following Surgery or Radiation for Prostate Cancer. Clin. Cancer Res..

[110] Ammar H.O., Shamma R.N., Elbatanony R.S.E., Khater B. (2020). Antioxidants in Cancer Therapy: Recent Trends in Application of Nanotechnology for Enhanced Delivery. Sci. Pharm..

[111] Cooke M., Evans M.D., Dizdaroglu M., Lunec J. (2003). Oxidative DNA damage: Mechanisms, mutation, and disease. FASEB J..

[112] Kumari S., Badana A.K., Malla R. (2018). Reactive Oxygen Species: A Key Constituent in Cancer Survival. Biomark. Insights.

[113] Pieniazek A., Czepas J., Piasecka-Zelga J., Gwozdzinski K., Koceva-Chyła A. (2013). Oxidative stress induced in rat liver by anticancer drugs doxorubicin, paclitaxel and docetaxel. Adv. Med. Sci..

[114] Khan H.Y., Zubair H., Ullah M.F., Ahmad A., Hadi S.M. (2012). A prooxidant mechanism for the anticancer and chemopreventive properties of plant polyphenols. Curr. Drug Targ..

[115] Azam S., Hadi N., Khan N., Hadi S. (2004). Prooxidant property of green tea polyphenols epicatechin and epigallocatechin-3-gallate: Implications for anticancer properties. Toxicol. Vitr..

[116] Zhong J., Rajaram N., Brizel D.M., Frees A.E., Ramanujam N., Batinic-Haberle I., Dewhirst M.W. (2013). Radiation induces aerobic glycolysis through reactive oxygen species. Radiother. Oncol..

[117] Forment J.V., O’Connor M.J. (2018). Targeting the replication stress response in cancer. Pharmacol. Ther..

[118] Greenlee H., Kwan M.L., Kushi L.H., Song J., Castillo A., Weltzien E., Quesenberry C.P., Caan B.J. (2011). Antioxidant supplement use after breast cancer diagnosis and mortality in the Life After Cancer Epidemiology (LACE) cohort. Cancer.

[119] Yang C.S., Luo P., Zeng Z., Wang H., Malafa M., Suh N. (2020). Vitamin E and cancer prevention: Studies with different forms of tocopherols and tocotrienols. Mol. Carcinog..

[120] Abrahamse H., Hamblin M.R. (2016). New photosensitizers for photodynamic therapy. Biochem. J..

[121] Vile G.F., Tyrrell R.M. (1995). Uva radiation-induced oxidative damage to lipids and proteins in vitro and in human skin fibroblasts is dependent on iron and singlet oxygen. Free. Radic. Biol. Med..

[122] Moss R.W. (2007). Do Antioxidants Interfere with Radiation Therapy for Cancer?. Integr. Cancer Ther..

[123] Broekgaarden M., Weijer R., Van Gulik T.M., Hamblin M.R., Heger M. (2015). Tumor cell survival pathways activated by photodynamic therapy: A molecular basis for pharmacological inhibition strategies. Cancer Metast. Rev..

[124] Jakus J., Farkas O. (2005). Photosensitizers and antioxidants: A way to new drugs?. Photochem. Photobiol. Sci..

[125] Naidoo C., Kruger C.A., Abrahamse H. (2019). Simultaneous Photodiagnosis and Photodynamic Treatment of Metastatic Melanoma. Molecules.

[126] Ishikawa T., Kajimoto Y., Sun W., Nakagawa H., Inoue Y., Ikegami Y., Miyatake S.-I., Kuroiwa T. (2013). Role of Nrf2 in Cancer Photodynamic Therapy: Regulation of Human ABC Transporter ABCG2. J. Pharm. Sci..

[127] Piette J. (2015). Signalling pathway activation by photodynamic therapy: NF-κB at the crossroad between oncology and immunology. Photochem. Photobiol. Sci..

[128] Tynga I.M., Abrahamse H. (2018). Nano-Mediated Photodynamic Therapy for Cancer: Enhancement of Cancer Specificity and Therapeutic Effects. Nanomaterials.

[129] Chung M.K., Kim D.H., Ahn Y.C., Choi J.Y., Kim E.H., Son Y.-I. (2016). Randomized Trial of Vitamin C/E Complex for Prevention of Radiation-Induced Xerostomia in Patients with Head and Neck Cancer. Otolaryngol. Neck Surg..

[130] Das A., Dey N., Ghosh A., Das S., Chattopadhyay D.J., Chatterjee I.B. (2012). Molecular and Cellular Mechanisms of Cigarette Smoke-Induced Myocardial Injury: Prevention by Vitamin C. PLoS ONE.

